# Prodigiosin as an Antibiofilm Agent against the Bacterial Biofilm-Associated Infection of *Pseudomonas aeruginosa*

**DOI:** 10.3390/pathogens13020145

**Published:** 2024-02-05

**Authors:** Zhiwen Ma, Hong Xiao, Hailin Li, Xiaoling Lu, Jing Yan, Hao Nie, Qi Yin

**Affiliations:** Department of Health Laboratory Technology, School of Public Health, Chongqing Medical University, No. 61 Daxuecheng Middle Road, Shapingba District, Chongqing 401334, China

**Keywords:** *Pseudomonas aeruginosa*, prodigiosin, anti-biofilm, chronic lung infection

## Abstract

*Pseudomonas aeruginosa* is known to generate bacterial biofilms that increase antibiotic resistance. With the increase of multi-drug resistance in recent years, the formulation of a new therapeutic strategy has seemed urgent. Preliminary findings show that Prodigiosin (PG), derived from chromium-resistant *Serratia marcescens*, exhibited efficient anti-biofilm activity against *Staphylococcus aureus*. However, its anti-biofilm activity against *P. aeruginosa* remains largely unexplored. The anti-biofilm activity of PG against three clinical single drug-resistant *P. aeruginosa* was evaluated using crystal violet staining, and the viability of biofilms and planktonic cells were also assessed. A model of chronic lung infection was constructed to test the in vivo antibiofilm activity of PG. The results showed that PG inhibited biofilm formation and effectively inhibited the production of pyocyanin and extracellular polysaccharides in vitro, as well as moderated the expression of interleukins (IL-1β, IL-6, IL-10) and tumor necrosis factor (TNF-α) in vivo, which might be attributed to the downregulation of biofilm-related genes such as *algA*, *pelA*, and *pslM*. These findings suggest that PG could be a potential treatment for drug-resistant *P aeruginosa* and chronic biofilm infections.

## 1. Introduction

Healthcare-associated infections (HAIs) pervade hospital settings and healthcare personnel and are attributable to the intricacy of the healthcare system; the prevalence of hospital-acquired or nosocomial infections exceeds 25% in developing countries and up to 15% in developed countries, resulting in the death of approximately 40,000 hospitalized patients worldwide [1]. *Pseudomonas aeruginosa* serves as a frequent causative agent of hospital-acquired Gram-negative bacilli infections, particularly affecting immunocompromised individuals. These infections commonly manifest in various forms, including respiratory, urinary tract, and bloodstream infections [2,3]. *P. aeruginosa* demonstrated sustained prominence as the most consistently clinically detected Gram-negative bacterium, as reported by the National Healthcare Safety Network (NHSN), spanning the years 2011 to 2017. It constituted a notable percentage, ranging from 16.5% to 32.6%, of all ventilator-associated bacterial infections in pneumonia, consistently ranking among the highest in detection rates. Furthermore, *P. aeruginosa* found itself included in the World Health Organization’s (WHO) list of microorganisms, requiring urgent attention for new antibiotic research in 2017 [4,5]. *P aeruginosa* employs two survival mechanisms: planktonic bacteria, inducing acute inflammation, and biofilm, leading to persistent infections. Biofilms, primarily composed of autogenic extracellular polymeric substances (EPSs), serve as a scaffold, encasing bacteria on surfaces and protecting them from environmental stresses [6]. Microbial biofilms irreversibly adhere to a biotic or abiotic surface that contributes to the protection of microorganisms against extreme conditions such as the environment, the administration of antibiotics and antifungals, and host immune mechanisms in response to infection [7]. The host’s immune response is triggered by the detection of various virulence factors of microorganisms, which are the necessary traits to establish an infectious process and interact directly with host cells [8]. The importance of studies on biofilm-forming strains lies in the increased resistance of these organisms to antimicrobial agents, the severity of infections that they can cause in humans and animals due to their difficult eradication, and the survival of microorganisms on abiotic surfaces like medical devices [9]. Biofilm antibiotic resistance is substantially higher (10 to 1000 times) than that of planktonic bacteria [10,11], and bacteria transitioning out of the biofilm state regain susceptibility to antibiotics [12]. Future research will likely focus on modulating bacterial biofilm status.

In the context of antibiotic replacement therapies targeting *P. aeruginosa* infections, antimicrobial peptides have been explored for their efficacy in inhibiting the Quorum Sensing (QS) system. However, their clinical application is hindered by high production costs and the potential for host cell hemolysis [13]. Furthermore, silica nanomaterials (SiNPs) show promise in preventing and eradicating biofilms, but their high toxicity poses a significant challenge [14]. Sterile crude supernatants derived from cultures of *Salmonella enterica* and *P. aeruginosa* have demonstrated effectiveness in reducing biofilm development in *P. aeruginosa* and *Klebsiella pneumonia*. Despite this, the specific active agents responsible for biofilm inhibition in these culture supernatants remain unidentified, necessitating further research [15]. The extraction of natural active compounds with bacteriostatic properties is underway, as these compounds are readily available and accessible. They are regarded as promising substitutes for conventional antibiotics in tackling the issue of bacterial resistance.

In a previous investigation, our research team isolated the *Serratia marcescens* CM01 strain and demonstrated its secondary metabolite prodigiosins (PG) as an effective agent in reducing drug-resistant *Staphylococcus aureus* biofilms [16] and modulating the intestinal immune response [17]. However, the specific impact of PG on *P. aeruginosa* biofilms remains unclear. In this study, a systematic investigation of the activities of PG against *P. aeruginosa* is conducted, including (i) detecting the antimicrobial and anti-biofilm activity against *P. aeruginosa* in vitro; (ii) determining the activity against chronic biofilm infection in vivo; (iii) profiling the phenotypic characteristics of these strains in vitro to preliminarily provide insights into the underlying antimicrobial mechanisms of PG.

## 2. Materials and Methods

### 2.1. Bacterial Strains and Culture Conditions

*Serratia marcescens* CM01 was isolated from the chromium-contaminated environment in the gathering area of a small and medium-sized electroplating factory in Chongqing, China [18]. The American Type Culture Collection (ATCC) provided the ATCC27853 and the PAO1; strains of β-lactam resistant *Pseudomonas aeruginosa* of CQMU105, CQMU184, CQMU293, CQMU308, CQMU359, and CQMU392 were isolated from patient secretions in a hospital’s secretion department (Chongqing, China) [19]. (Please see the Supplementary Materials for specific information about the strains.)

*P. aeruginosa* was incubated on Lysogeny broth (LB, Hope Bio-Technology Co., Ltd., Qingdao, Shandong, China) medium for 24 h at 37 °C and *S. marcescens* CM01 was incubated on LB liquid medium for 24 h at 30 °C.

### 2.2. Mice Ethics Statement

Male C57BL/6J mice aged six to eight weeks and weighing between 20 and 25 g (SCXK2019-0004, Slake Jinda Laboratory Animal Co. Ltd., Changsha, Hunan, China) were housed in the Chongqing Medical University Animal Center. The mice gradually adapted to the experimental cage settings (23 °C temperature, 55 °F humidity, and 12/12 h light-dark cycle) for one week. They were randomly divided into 4 groups (n = 8) according to the experimental design. Animal experiments were approved by the Ethics Committee of the Chongqing Medical University (IACUC-CQMU-2023-03012) and carried out in compliance with the institutional guidelines concerning animal use and care of Chongqing Medical University.

### 2.3. Isolation, Purification, Characterization, and Quantification of Prodigiosin (PG)

For *S. marcescens* CM01 subculturing, single colonies were selected into LB broth and shaken overnight at 37 °C. Then, the overnight bacterial culture was diluted 1:100 to introduce it into the broth, and it was allowed to develop for 48 h at 30 °C before being centrifuged to remove the bacteria. For extraction, the necessary volume of acidic methanol (pH = 3.0) was combined with the bacterial precipitate. An ultrasonic cell crusher (SCIENTZ-IID, Ningbo, Zhejiang, China) was used to sonicate the mixture for 10 to 30 min at intervals of 5 s. Throughout sonication, an ice bath was maintained to ensure consistent temperature control. The mixture was sonicated and then centrifuged for 5 min at a speed of 12,000× *g* to remove all remaining pigment from the precipitate. The supernatant’s crude PG extract was poured into a new tube. By using silica gel column chromatography, the unpurified PG extract was refined. Ethyl acetate and acetone were used as the eluent at a ratio of 1:1 (*v*/*v*). To remove the eluent, the purified PG was put in a flask with a circular bottom and fitted on a rotating evaporator at 37 °C. The dried PG was reconstituted in the essential volume of acidic methanol (pH = 3.0, chromatographic purity, purity ≥ 99.9%, Tiandi High Purity Solvent Co., Ltd., Anqing, Anhui, China) and then passed through a 0.22 μm filter membrane. We used high-performance liquid chromatography (HPLC) to verify the presence and purity of PG, and we used a Sepax Bio-C18 column (4.6 × 150 mm × 3μm, Agilent Technologies, Santa Clara, California, USA) with HPLC (1260 Infinity II) for 20 min with an injection volume of 10 μL of crude PG extract. The mobile phase was 7:3 (*v*/*v*) methanol: water (pH = 3.0) and its detection wavelength was 535 nm [20].

### 2.4. In Vitro Antimicrobial Activity Assay

The antimicrobial activity of PG and the antibiotic susceptibility of PAO1 and clinical *P. aeruginosa* isolates were determined according to CLSI guideline M100-S28 [21]. The trials were repeated six times separately.

### 2.5. In Vitro Biofilm Formation Inhibitory Activity Assay

Crystal violet (CV, Sangon Bioengineering Co., Ltd., Shanghai, China) [22] was used to calculate the minimal biofilm inhibitory concentration assay. Each strain’s overnight cultures were diluted to 5 × 10^6^ CFU/mL in LB containing 2% glucose [23], and then 2 μL of PG was dissolved in acidic methanol (pH = 3.0) at final concentrations of 1, 5, 10, 15, 20 μg/mL (PAO1) and 1, 2, 4, 6 μg/mL (CQMU293, CQMU308, CQMU359) were added into each well of the 96-well plates (NEST, Life Science and Technology Co., Ltd., Wuxi, Jiangsu, China). Then, the plates were incubated at 37 °C for 24 h. Acidic methanol (pH = 3.0) was used as a negative control, with 2 μL added into each well. Following incubation, planktonic bacteria were eliminated using 1 × PBS. Each well was filled with a 0.1% crystal violet solution and stained for 10 min at room temperature. Excess crystal violet was washed away with sterile water, allowed to air dry, and subsequently reconstituted using anhydrous ethanol, and then the OD595 nm was measured by a microplate reader (Thermo Fisher Scientific Inc., Waltham, MA, USA) [16]. The minimum biofilm inhibitory concentration (MBIC) is the lowest concentration of the antimicrobial agent to inhibit the initial formation of biofilm, indicated by no color development.

### 2.6. Bacteria Counts of Biofilms

Colony Forming Unit (CFU) counts were employed to assess the viability of bacterial cells within the biofilm. Acidic methanol (pH = 3.0) was used as a negative control, with 2 μL added into each well. Following the MBIC experiments, the biofilms were washed twice with 1 × PBS and then resuspended in 1 mL of sterile saline solution. Subsequently, the biofilm cells were serially diluted 10-fold and 100 μL of each dilution was plated onto LB agar, followed by a 24-h incubation period for CFU/mL enumeration. This experiment was repeated three times.

### 2.7. Extraction of Pyocyanin, Extracellular Polysaccharide, and Alginate

The treatment groups received additions of MBIC and 1/2 minimum inhibitory concentration (MIC) quantities of PG, respectively. Acidic methanol (pH = 3.0) was used as a negative control, with 2 μL added into each well.

Pyocyanin (PCA) was used with the hydrochloric acid–chloroform technique to extract [24], and it was measured at OD_520 nm_ at 3, 6, 9, 12, and 24 h after incubation.

Extracellular polysaccharide (EPS) was detected using the phenol–sulfuric acid method [24]. After incubation, the absorbance of the mixture was measured at 490 nm.

The methods of Yasuda H et al. [25] were consulted for the alginate extraction. After that, the material was washed twice with 20% NaCl and determined at OD_565 nm_.

All experiments were repeated three times.

### 2.8. Real-Time PCR Analysis

The Simple P Total RNA Extraction Kit (Hangzhou Bioer Technology Co, Ltd., Hangzhou, Zhejiang, China) with PG concentrations of 16 μg/mL (1/2MIC) and 1 μg/mL (MBIC) was used. Acidic methanol (pH = 3.0) was used as a negative control, with 2 μL added into each well. The total RNA from all groups was extracted. Following the creation of cDNA using the Prime Script TM RT kit (Takara, Otsu, Shiga, Japan), the expression of the biofilm-related genes *algA*, *pelA*, and *pslM* was detected using real-time fluorescence real-time PCR (RT-PCR). The cycling thresholds (Ct) of each detected gene were normalized using *proC* as the internal reference gene and quantified using the 2^−ΔΔct^ method relative expression. Table 1 contains the RT-PCR primer sequences [26] that were employed. The experiments were repeated three times.

### 2.9. Chronic Lung Infection of Mice Model

For the chronic biofilm infection model, 1.0 × 10^6^ CFU/mL of agar bead embedded PAO1 in 50 μL was intratracheally instilled into the lungs of mice [27]. After 2 h of infection, allowing initial attachment for biofilm formation, the mice were treated with PG (200 μg/kg) [28]. PBS was used as a negative control, and aztreonam (AZT) at 20 mg/kg was used as a positive control [19]. After 24 h [29] of treatment, whole lungs were aseptically removed and a slice of a pulmonary lobe from each mouse was used for histological examination haematoxylin and eosin (H&E) staining [30]. Images were acquired using a microscope (Olympus BX53F2, Tokyo, Japan). Approximately ~0.1 g of lung tissue was aseptically excised and homogenized in sterile saline for bacterial load enumeration and the residual lung tissue was used to detect inflammatory factors. Lung tissue samples were frozen in liquid nitrogen and mechanically dissociated in an RNA buffer. Total RNA was extracted and cDNA was obtained by reverse transcription using Trizol reagent (Molecular Research Center, Inc., Cincinnati, OH, USA) according to the manufacturer’s protocol. cDNA samples were diluted twice and used as amplification templates. Each sample was added to 3 wells and run in a Quant Studio 6 Flex RT-qPCR system. Relative expression levels of genes were calculated with the 2^−ΔΔct^ method by using reference gene GAPDH to normalize. The RT-PCR primer sequences [17] used are shown in Table 2.

### 2.10. Statistical Analysis

Multiple comparisons between groups were analyzed by using one-way ANOVA in GraphPad Prism (GraphPad Prism 8.0.1, GraphPad, San Diego, CA, USA), followed by Tukey’s test. *p* values < 0.05 were considered significant.

## 3. Results

### 3.1. Qualitative and Quantitative Investigation of Prodigiosin (PG)

The High Performance Liquid Chromatography (HPLC) results showed that PG was present in the secondary metabolites of *Serratia marcescens* CM01, with both the PG standard and the crude extract having a single maximum absorption peak at 535 nm. Chromatograms were derived from the group’s initial data [16,17] (Supplementary Materials).

### 3.2. PG Showed Antimicrobial Activity against β-Lactam-Resistant Pseudomonas aeruginosa

The susceptibility results (minimum inhibitory concentration values, MICs) for PAO1 and clinical *P. aeruginosa* are shown in Table 3. The MIC values of PG for clinical *P. aeruginosa* ranged from 8 to 64 μg/mL, with MIC values of 32 μg/mL for strains PAO1. Notably, when PG was 8 to 16 μg/mL, it inhibited five β-lactam-resistant *P. aeruginosa* strains; 32 μg/mL killed four β-lactam-resistant strains, and 128 μg/mL killed six β-lactam-resistant strains (which are the minimum bactericidal concentration values, MBCs) (Table 3), indicating that *P. aeruginosa* was sensitive to PG.

### 3.3. PG Exhibited Biofilm Inhibitory Activities against P. aeruginosa In Vitro

**Figure 1:**
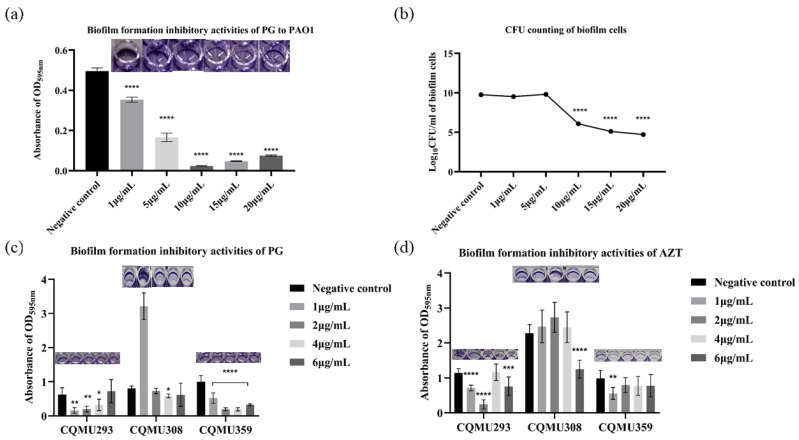
Anti-biofilm activity of PG on *P. aeruginosa* biofilm formation. (**a**) Anti-biofilm activity of PG on PAO1 of the biofilm formation. (**b**) CFU counting of biofilm after minimum biofilm inhibitory concentration (MBIC) assay of PG on PAO1 for 24 h. (**c**,**d**) Anti-biofilm activity of PG or AZT on CQMU293, CQMU308, and CQMU359 of the biofilm formation. Bacteria were exposed to different concentrations of PG or AZT and incubated at 37 °C for 24 h to detect the efficiency of inhibition of their biofilm formation; acidic methanol was used as negative control, with 2 μL added into each well. Subsequent procedures followed the same protocol unless otherwise specified (MBIC assay). * represents *p* < 0.05; ** represents *p* < 0.01; *** represents *p* < 0.001, **** represents *p* < 0.0001.

In vitro, anti-biofilm activity assays revealed that MBIC values of PG ranged from 1 to 4 μg/mL against PAO1 and strains CQMU293, CQMU308, and CQMU359. Under 1 μg/mL PG, the biofilm biomass was reduced by 18%, 74%, and 53% for strains PAO1, CQMU293, and CQMU359, respectively (Figure 1a,c). But with the same dose of aztreonam (AZT), the biofilm biomass was only reduced by 36% and 43% for strains CQMU293 and CQMU359 (Figure 1d). For PAO1, the viable cell count in the biofilm displayed no significant variations with gradually increasing concentrations of PG, while the reduction in biofilm biomass showed a PG concentration-dependent effect (Figure 1a,b), indicating that PG might inhibit biofilm formation by targeting specific biofilm-related targets instead of simply killing bacteria.

### 3.4. PG Decreased the Content of Pyocyanin (PCA), Alginate, and Extracellular Polysaccharide (EPS)

**Figure 2:**
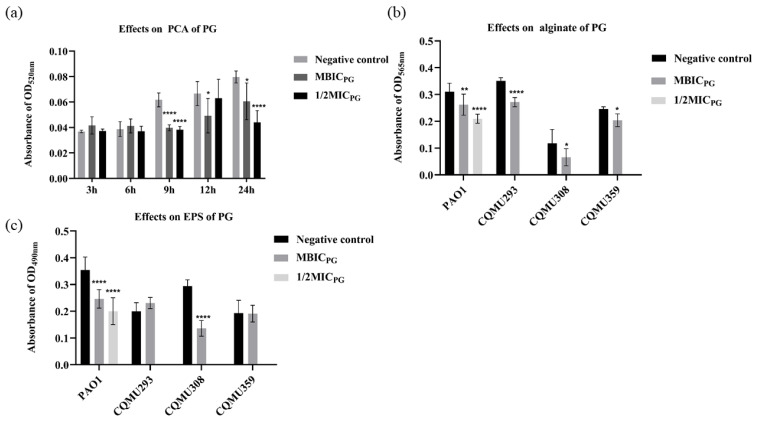
(**a**) Production of pyocyanin (PCA) at 3, 6, 9, 12, and 24 h of PG intervention on PAO1; (**b**) production of alginate after 24 h of PG intervention on PAO1; (**c**) production of extracellular polysaccharide (EPS) after 24 h of PG intervention on PAO1. Acidic methanol was used as negative control, with 2 μL added into each well. MBIC_PG_ = 1 μg/mL 1/2MIC = 16 μg/mL. * represents *p* < 0.05; ** represents *p* < 0.01; **** represents *p* < 0.0001.

To explore the mode of action of PG against the biofilm formation of *P. aeruginosa*, the effect of PG was determined on the content of PCA, alginate, and EPS in the biofilms of PAO1, CQMU293, CQMU308, and CQMU359. After treatment with PG under MBIC and 1/2MIC for 24 h, the formation of PCA was reduced by 33% and 95%, alginate by 15% and 32%, and EPS by 29% and 48% in PAO1, respectively (Figure 2a–c). After treatment with PG under MBIC for 24 h, the formation of EPS and alginate in CQMU308 was reduced by 44% and 53%. At the same time, the formation of EPS and alginate in CQMU359 was reduced by 17% and 1%. At the same condition, the formation of alginate in CQMU293 was reduced by 22%, but the EPS was increased by 13%. This suggests that PG effectively prevented the synthesis of PCA, alginate, and EPS, reduced the virulence effect of bacteria, and allowed bacteria to scatter without adhering or aggregating.

### 3.5. Effects of PG on the Gene Expression of EPS

**Figure 3:**
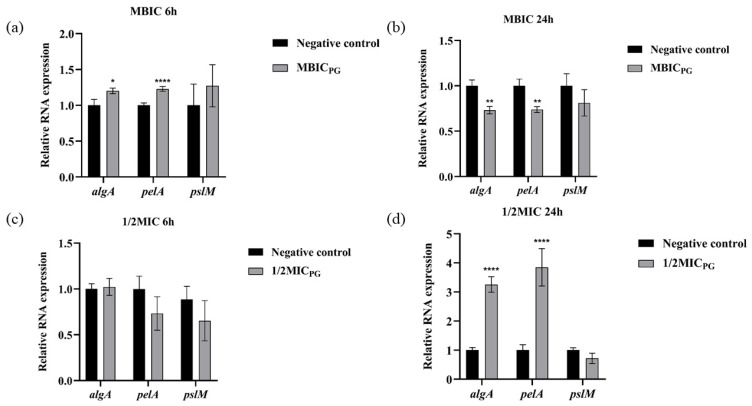
Effect on the expression of the PAO1 biofilm-related genes *algA*, *pelA,* and *pslM* at 6 h (**a**,**c**) and 24 h (**b**,**d**) under MBIC (1 μg/mL) and 1/2 MIC (16 μg/mL) of PG. The gene of *proC* was used as a reference gene. Acidic methanol was used as negative control, with 2 μL added into each well. * represents *p* < 0.05; ** represents *p* < 0.01, **** represents *p* < 0.0001.

To further investigate the mechanism of action (MOA) of PG against biofilm formation, several genes related to the production of EPS were selected to detect the expressed level under PG exposure. The expression of three important transcription factors was downregulated after PG exposure at MBIC for 24 h by PAO1 (Figure 3b), which replicated the prior phenotypic results. Curiously, *pelA* and *pslM* were downregulated after PG exposure at 1/2MIC for 24 h (Figure 3c).

### 3.6. The Effects of PG on Chronic Lung Infection in Mice

#### 3.6.1. In Vivo Anti-Biofilm Activity of PG

**Figure 4:**
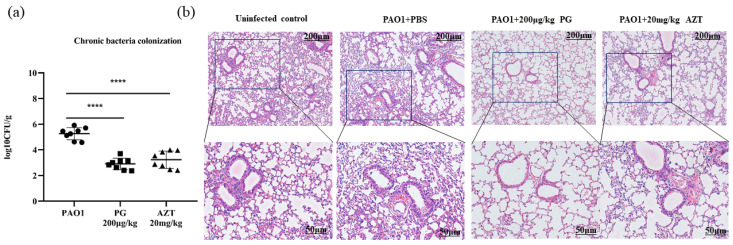
(**a**) In the chronic biofilm infection mouse model with an initial bacterial load of 1 × 10^4^ CFU of PAO1, bacterial colonization of the lung was harvested after 24 h of PG or AZT treatment to conduct CFU counting (n = 8). Among them, symbols represent the individual value of each group **** represents *p* < 0.0001. (**b**) Photomicrographs (100%, bar 200 μm) of H&E staining of the lungs in the chronic infection model. The large boxed areas show higher-magnification views (400%, bar 50 μm) of the small boxes.

In the chronic biofilm infections, the bacterial loads in the lung were controlled at 1 × 10^4^ CFU/g in the 200 μg/kg PG group compared with 1 × 10^6^ CFU/g in the negative control group (Figure 4a). At the same time point, the PAO1 group showed more significant levels of inflammation in the lungs (Figure 4b), expanded alveoli, and inflammation infiltration throughout the airways and alveolar walls than the uninfected group. In contrast, tail vein injections of PG and AZT reduced inflammation in the lungs of mice, with a significant decrease in inflammatory infiltration around the walls of the airways and alveoli. The H&E results revealed low inflammatory damage to lung tissue by PG, which was in keeping with the lower lung residual bacterial load. In conclusion, PG was able to inhibit chronic lung infections caused by *P. aeruginosa.*

#### 3.6.2. Effect of PG on Mice Lungs of Inflammatory Factors

**Figure 5:**
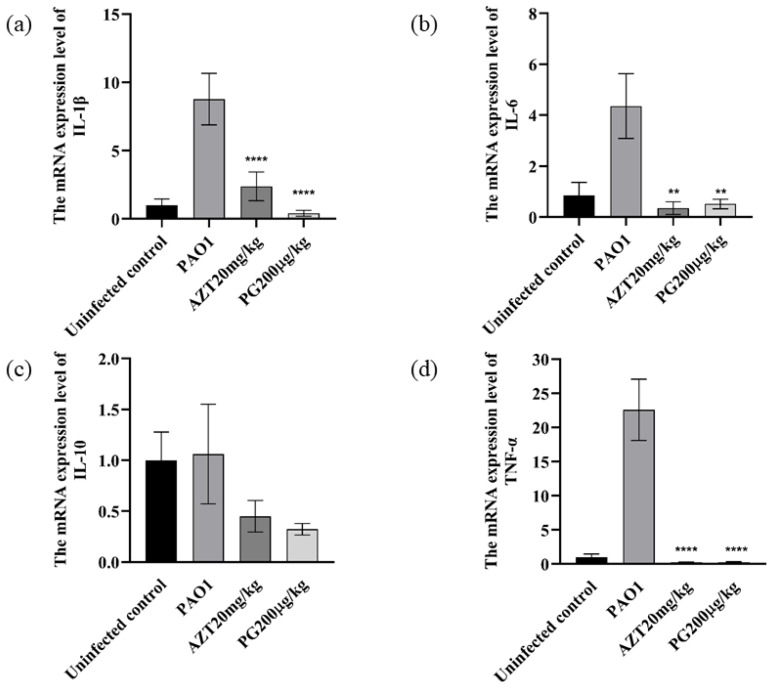
Effects of PG on inflammatory factors: IL-1β (a), IL-6 (b), IL-10 (c), and tumor necrosis factor: TNF-α (d) of C57BL/6J mice with chronic infection. ** represents *p* < 0.01; **** represents *p* < 0.0001.

RT-PCR was used to measure the levels of interleukin-1 (IL-1β), interleukin-6 (IL-6), interleukin-10 (IL-10), and tumor necrosis factor (TNF-α) in the lungs to determine the impact of PG on cytokine production. IL-1β, IL-6, and TNF-α levels were noticeably higher in the PAO1 group compared to the control group, as shown in Figure 5 (*p* < 0.01). However, pretreatment with PG and AZT significantly decreased these increments (*p* < 0.01). Additionally, the levels of IL-10 in the PG and AZT pretreatment groups were also lower than those in the PAO1 group (*p* > 0.05), indicating that the mice in the PAO1 group had higher levels of IL-10 expression, which may indicate that macrophages secrete a lot of IL-10 to reduce inflammatory responses. The aforementioned findings imply that PG controls pulmonary cytokine production in a mouse model of chronic *P. aeruginosa* infection.

## 4. Discussion

Due to the widespread role of *Pseudomonas aeruginosa* in causing various infections and increasing antibiotic resistance, treatment failure has become a major global problem. *P. aeruginosa* has shown high intrinsic resistance to a range of antibiotics, including beta-lactams, fluoroquinolones, and aminoglycosides, which results in significant morbidity and mortality rates [31,32]. According to a U.S. Centers for Disease Control and Prevention report, it is estimated that approximately 51,000 healthcare-associated infections caused by *P. aeruginosa* occur in the United States each year, and 13% of these infections are multidrug-resistant (MDR), with roughly 400 million deaths per year attributed to such infections [33,34]. The primary mechanisms underlying antibiotic resistance include the low permeability of the outer membrane, chromosomally encoded AmpC, and drug efflux through multi-drug efflux (Mex) systems [35]. Within these contexts, microbial communities known as biofilms, composed of extracellular proteins, polysaccharides, and nucleic acids [36], play a pivotal role. Bacteria residing in biofilms exhibit resistance to both the human immune system and pharmaceutical interventions, leading to the development of chronic infections [37]. Consequently, strategies aimed at preventing the formation of biofilms and maintaining bacteria in a planktonic state represent potentially valuable approaches.

In the prior study, the isolation of the *Serratia marcescens* CM01 strain unveiled its secondary metabolite, prodigiosin (PG), exhibiting potent efficacy in reducing drug-resistant *S. aureus* biofilms [16]. Additionally, the study demonstrated the ability of PG to modulate the immune response within the intestinal environment [17]. Among reported anti-biofilm agents, PG produced from *Serratia* sp. C6LB significantly inhibited the strain of *S. aureus* [38]. With dose and time-dependent inhibitory effects, PG dramatically suppressed the development of human choriocarcinoma (JEG3) and prostate cancer (PC3) cells [39]. Additionally, PG exhibited preventive properties against the proliferation of reactive oxygen species, known for biomolecule degradation, thereby impeding *P. aeruginosa* biofilm formation [40].

Despite extensive prior investigations into the efficacy of PG, the precise mechanism underlying the inhibition of *P. aeruginosa* biofilms by PG remains elusive. The present study emphasized PG as a promising pharmaceutical candidate with the ability to hinder clinical infections linked to *P. aeruginosa* and impede biofilm formation. In the initial phase, we assessed the minimum inhibitory concentration values (MICs) of PG against six β-lactam-resistant strains of *P. aeruginosa*, revealing that five of them exhibited MICs lower than PAO1 (64 μg/mL), indicating the efficient antimicrobial activity of PG. Subsequently, we investigated the in vitro anti-biofilm activity of PG against *P. aeruginosa*. PG demonstrated a biofilm inhibitory effect ranging from 27% to 74% at concentrations of 1 to 4 μg/mL (equivalent to 1/4 to 1/2 of its MIC) against β-lactam-resistant strains of *P. aeruginosa* and an 18% biofilm inhibitory effect at 1 μg/mL against PAO1. Interestingly, the minimum biofilm inhibitory concentration (MBIC) of PG did not exhibit bactericidal efficacy against PAO1 but demonstrated a dose-dependent impact on biofilm development; it appears that PG may achieve the inhibition of biofilm formation by modulating specific genes to suppress the production of extracellular polysaccharides, rather than directly targeting or killing bacteria. To contextualize the effectiveness of PG, comparisons were made with other anti-biofilm agents. For instance, butenolide, recognized as a broad-spectrum anti-biofilm agent against *P. aeruginosa*, exhibited an MBIC of 800 μg/mL [41]. Isoprenaline demonstrated efficacy against *Burkholderia pseudomallei* biofilms with Minimum Biofilm Eradication Concentrations (MBECs) ranging from 780 to 3120 μg/mL [42]. Additionally, romelinic acid, at a concentration of 1000 μg/mL, resulted in a 70% reduction in the biofilm of *Aeromonas hydrophila* [43].

The findings demonstrated that PG reduced both biofilm and planktonic cell counts of *P. aeruginosa*. Previous data have established PG as a hydrophobic stressor, causing damage to the bacterial plasma membrane via a dissociation-mediated mechanism. This damage impedes bacterial growth, resulting in the leakage of intracellular material [44]. In light of these results, PG exhibited notable bacteriostatic and anti-biofilm efficacy against *P. aeruginosa*, with the added capability of eliminating planktonic bacteria at specific doses.

Regarding its clinical potential, PG was reported to impact intestinal flora and aid in the treatment of gastrointestinal diseases [17]. Results from our model of chronic lung infection indicated that 200 μg/kg of PG exert immunomodulatory effects on lung infection compared to a negative control. PG reduced the bacterial load and expression levels of IL-1β, IL-6, IL-10, and TNF-α in the lungs of mice. And it ensured the typical functioning of organisms in terms of expelling pathogenic bacteria and toxins, controlling inflammatory responses, and preventing waterfall effects and severe infections.

In the discussion of the Mechanism of Action (MOA) of PG, our focus centered on the study of extracellular polysaccharide (EPS) and three associated genes. EPS, composed of embedded proteins, plays a crucial role in determining biofilm morphology, offering protection against adverse environmental conditions and contributing to biofilm viscoelasticity [45]. *P. aeruginosa* produces at least three types of EPS, namely: (i) alginate, a polysaccharide consisting of mannuronic and guluronic acid; (ii) a glucose-rich exopolysaccharide synthesized by enzymes encoded by the *pel* gene cluster; and (iii) a mannose-glucose polysaccharide produced by proteins encoded in the *psl* gene cluster [46,47,48]. Each of these polysaccharides is associated with distinct stages of *P. aeruginosa* biofilm development. Alginate, the first discovered *P. aeruginosa* exopolysaccharide, is linked to the “mucoid” phenotype of strains isolated from cystic fibrosis (CF) patients [49]. It provides chemical protection against antibiotics and immunological responses [50]. Pel contributes to antibiotic resistance [51], while Psl protects biofilms from drugs through chemical binding and aids in adhesion to solid surfaces [52]. PG was found to influence the expression of *sarA* and *agrA* by regulating the global regulatory factor *sigB*, known to inhibit biofilm formation [16]. This aligns with the outcomes of our study against *P. aeruginosa*. In this investigation, we ascertained that EPS might be the target of PG action. It was observed that PG can reduce EPS production by downregulating *algA, pelA*, and *pslM* genes, thereby reducing bacterial adhesion and aggregation in biofilm structures. This regulatory effect helps the bacteria return to a planktonic state, thereby significantly enhancing drug sensitivity. However, whether the reduction in biofilm amount is due to the inhibition of microbial growth or early inhibition of biofilm formation requires further exploration. It is noteworthy that following 24 h of MBIC exposure to PG, the clinical strain CQMU293 exhibited a decrease in alginate content and an increase in EPS content. This phenomenon could be attributed to the speculated concept that reduced alginate expression might enhance the attachment of planktonic bacteria and the biofilm matrix, subsequently leading to an increase in EPS [53]. However, it is essential to acknowledge that this interpretation is speculative, and further investigation is warranted to delve deeper into the underlying mechanisms.

Taken together, our preliminary results in vitro and in vivo indicated the underlying mode of action of PG in combating *P. aeruginosa* biofilm in three ways: (i) reducing the production of EPS, decreasing the quantity of bacterial biofilm, and impairing the protection of bacteria biofilm to bacterial cells against the environment; (ii) relying on its antibacterial activity to reduce bacterial loading; and (iii) complementarily modulating the immune system response, leading to efficient anti-infection and anti-inflammatory activities.

Finally, this study acknowledges several limitations: (i) Further detailed molecular experiments are warranted to elucidate the mechanism by which PG inhibits biofilm formation, aiding in PG’s potential to counteract the resistance mechanisms of *P. aeruginosa.* (ii) Subsequent research should focus on preventing *P. aeruginosa* biofilm infections and conducting combination experiments with antibiotics, facilitating a more comprehensive understanding to guide the clinical application of PG.

## 5. Conclusions

In summary, our study illustrated that prodigiosin (PG) generated by *Serratia marcescens* CM01 is a potent antimicrobial agent against β-lactam-resistant *Pseudomonas aeruginosa* and chronic infections associated with biofilms. We outlined the potential antimicrobial mechanism of PG, suggesting its interaction with extracellular polysaccharide EPS in the outer membrane. This research underscored the promise of PG as a candidate drug for the treatment of clinical *P. aeruginosa* and chronic infections related to biofilms.

## Figures and Tables

**Table 1:** List of primers of the extracellular polysaccharide in real-time PCR (RT-PCR) reaction.

Target Name	Type	Primer Sequences (5′ to 3′)
*proC*	Fw	CAGGCCGGGCAGTTGCTGTC
	Rev	GGTCAGGCGCGAGGCTGTCT
*algA*	Fw	AGAACTGAAGAAGCACGACG
	Rev	TTCTCCATCACCGCGTAGT
*pelA*	Fw	ATGGCTGAAGGTATGGCTG
	Rev	AGGTGCTGGAGGACTTCATC
*pslM*	Fw	CTATGACGCACGGCAACTGG
	Rev	CGCCATTGACCAGGTGCAT

**Table 2:** List of primers of the inflammatory factors and tumor necrosis factor in RT-PCR reaction.

Target Name	Type	Primer Sequences (5′ to 3′)
GADPH	Fw	GGACTTACAGAGGTCCGCTT
	Rev	CTATAGGGCCTGGGTCAGTG
IL-1β	Fw	CAACCAACAAGTGATATTCTCCATG
	Rev	GATCCACACTCTCCAGCTGCA
IL-6	Fw	GAGGATACCACTCCCAACAGACC
	Rev	AAGTGCATCATCGTTGTTCATACA
IL-10	Fw	CGAGATGCCTTCAGCAGAG
	Rev	CGCCTTGATGTCTGGGTCTT
TNF-α	Fw	CATCTTCTCAAAATTCGAGTGACAA
	Rev	TGGGAGTAGACAAGGTACAACCC

**Table 3:** The minimum inhibitory concentration (MIC) and minimum bactericidal concentration (MBC) values of PG and AZT against *P. aeruginosa*.

Strains	MIC_PG_ (μg/mL)	MBC_PG_ (μg/mL)	MIC_AZT_ (μg/mL)	MBC_AZT_ (μg/mL)
PAO1	32	128	-	-
CQMU105	64	128	128	256
CQMU184	16	32	256	>256
CQMU293	8	32	32	128
CQMU308	8	32	64	>128
CQMU359	8	32	32	64
CQMU392	16	32	128	256

MIC_PG_ and MBC_PG_ present the minimum inhibitory concentration (MIC) and minimum bactericidal concentration (MBC) values of PG; MIC_AZT_ and MBC_AZT_ present the minimum inhibitory concentration (MIC) and minimum bactericidal concentration (MBC) values of AZT.

## Data Availability

The data that support the findings of this study are available from the corresponding author upon reasonable request.

## Supplementary Materials

The following supporting information can be downloaded at: https://www.mdpi.com/article/10.3390/pathogens13020145/s1, File S1: The storage number of *Serratia marcescens* CM01; Table S1: Clinical isolates of *Pseudomonas aeruginosa* used in this study, and the drug resistant description; Figure S1: The chromatograms of prodigiosin.
