# Supplementary material for: Prodigiosin as an Antibiofilm Agent against the Bacterial Biofilm-Associated Infection of Pseudomonas aeruginosa
Source: Pathogens. 2024 Feb 5;13(2):145. doi: 10.3390/pathogens13020145 (PMC10891946; doi:10.3390/pathogens13020145)
Supplement: Supplementary file 1 [file pathogens-13-00145-s001.zip › Supplementary Figure S1. The chromatograms of prodigiosin.pdf]

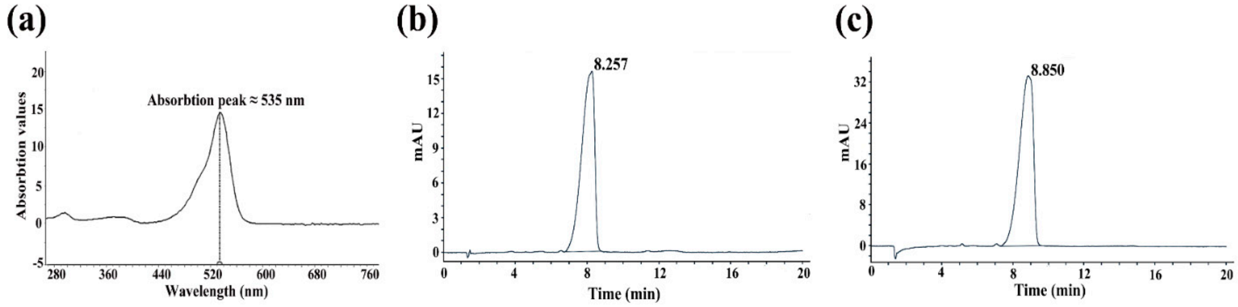

Supplementary figure S1. (a) The maximum absorbance of the extracted prodigiosin sample was obtained at 535 nm; (b) Chromatogram of purified prodigiosin sample; (c) Chromatogram of 20 μg/mL prodigiosin standard.
