# Supplementary material for: Prodigiosin as an Antibiofilm Agent against the Bacterial Biofilm-Associated Infection of Pseudomonas aeruginosa
Source: Pathogens. 2024 Feb 5;13(2):145. doi: 10.3390/pathogens13020145 (PMC10891946; doi:10.3390/pathogens13020145)
Supplement: Supplementary file 1 [file pathogens-13-00145-s001.zip › Supplementary File S1. The storage number of Serratia marcescens CM01.pdf]

中国海洋微生物菌种保藏管理中心
Marine Culture Collection of China

CERTIFICATE OF DEPOSIT

IN MARINE CULTURE COLLECTION OF CHINA

Marine Culture Collection of China
Third Institute of Oceanography, Ministry of Natural Resources
No. 178 Daxue Road, 361005 Xiamen, Fujian Province
P. R. China.
Phone/Fax: +86-592-2195177
Email: mccc5177@163.com
Web site: http://www.mccc.org.cn

MCCC 1K08654

Serratia **sp. (strain CM01) was received for deposit**
in Marine Culture Collection of China from

hong Xiao

College of Public Health, Chongqing Medical University
No. 61, College Town Mid. Rd, ShaPingBa District, Chongqing
P. R. China

on Mar. 22, 2023

and was, after confirming the viability and purity,
allocated the accession number MCCC 1K08654.

The strain is available to any bona fide scientific community or individual,
operating in a professional environment
suitable for handling living material of the biohazard group involved.

Xiamen, May 16, 2023

Dr. Zongze Shao
Public Collection Curator
Marine Culture Collection of China
